# The utilization of multidisciplinary tumor boards (MDT) in clinical routine: results of a health care research study focusing on patients with metastasized colorectal cancer

**DOI:** 10.1007/s00384-017-2871-z

**Published:** 2017-08-05

**Authors:** Markus Lowes, Mathias Kleiss, Rainer Lueck, Sven Detken, Alexander Koenig, Manuel Nietert, Tim Beissbarth, Kathrin Stanek, Claus Langer, Michael Ghadimi, Lena-Christin Conradi, Kia Homayounfar

**Affiliations:** 1 Department of General, Visceral and Pediatric Surgery, University Medical Center Georg-August-University, Robert-Koch-Strasse 40, 37075 Göttingen, Germany; 2 Department of Interdisciplinary Oncology, Red Cross Hospital Kassel, Kassel, Germany; 3 Department of General and Visceral Surgery, Sana-Klinikum Hameln-Pyrmont, Hameln, Germany; 4 Outpatient Clinic for Hematology and Oncology Northeim, Northeim, Germany; 5 Department of Gastroenterology and Gastrointestinal Oncology, University Medical Center Goettingen, Goettingen, Germany; 6 Department of Medical Statistics, University Medical Center Goettingen, Goettingen, Germany; 7 Department of General, Visceral, Thoracic and Minimal-Invasive Surgery, Protestant Hospital Weende, Goettingen, Germany; 8 Department of General, Visceral and Endocrine Surgery, Red Cross Hospital Kassel, Kassel, Germany

**Keywords:** Multidisciplinary tumor board, Health care research, Metastasized colorectal cancer

## Abstract

**Purpose:**

Multidisciplinary tumor boards (MDT) have been advocated as standard of care in modern oncology. German guidelines for metastasized colorectal cancer (mCRC) recommend MDT discussion of colon cancer patients after completion of primary tumor therapy but stage IV colon cancer as well as rectal cancer patients prior to any therapy. In this health care research study, we evaluated application and decisional consequences of this approach in clinical routine.

**Methods:**

All major institutions providing oncological care in southern Lower Saxony and Northern Hesse (*N* = 11) were invited. Patients with mCRC diagnosed between 01/2011 and 12/2013 were eligible. Data were collected using a standardized patient report form and stored in a GCP-conform EDC-system (secuTrial®).

**Results:**

A university medical center, four teaching hospitals, one communal hospital, and three oncological focus practices participated in the study. In total, 470 patients with a median age of 70 years were enrolled. Guideline conform MDT discussion was performed in 63% of operated colon cancer patients, 38% of stage IV colon cancer patients and 47% of rectal cancer patients, respectively. Resection of metastases was performed in 41% of cases. Patients ≥70 years (*n* = 250) received significantly more often treatment following MDT discussion (86 versus 64%, *p* = 0.0002). Not the resection rate (48 versus 57%, *p* = 0.1574) but indication for preoperative chemotherapy (57 versus 33%, *p* = 0.0056) significantly differed when patients with single organ metastases experienced MDT discussion.

**Conclusions:**

MDT discussion is not as established as advocated by national guidelines. Treatment decisions differ especially in older patients and those with single organ metastases.

## Introduction

Cancer is a leading cause of death worldwide [1]. However, in the last 2 decades, numerous new treatment options including newly developed drugs, antibodies, and different local ablative therapies as well as extended indication for resection in metastatic disease have been implemented in clinical routine extending the armentarium to preserve quality of life and/or even prolong disease free (DFS) or overall survival (OS) in cancer patients. As a consequence of these numerous options and improvements, multidisciplinary tumor boards (MDT) have been advocated as standard of care in modern oncology. Recent national cancer guidelines demand pretherapeutical discussion of every cancer patient in these MDT [2–4].

In order to improve cancer care in Germany, the Federal Ministry of Health, the German Cancer Society, the German Cancer Aid and the Working Committee of German Cancer Centers have initiated the National Cancer Act in 2008, a collection of numerous future goals and recommendations in all fields of cancer care including screening, quality improvement, cross-sectoral cooperation, and documentation [5]. In addition to the above described medical perspective of MDT necessity, this National Cancer Act argues not from the individual but from the societal perspective and demands high quality cancer care for every patient irrespective of age, sex, origin, habitation, and state of insurance. This goal should be reached by promoting high-quality oncological care on the one hand and reduction of inacceptable discrepancies in health care quality between regions on the other hand and thereby development of suitable health care structures including MDT.

Given that knowledge and attitude are not limited factors in the health care system of a developed country such as Germany, the aim of the present health care research study was to evaluate real life performance in terms of MDT discussion of cancer patients within a defined but representative region and focused on a frequent cancer type, namely metastasized colorectal cancer (mCRC). For this cancer, the German guidelines recommend to discuss every patient with colon cancer (CC) after primary tumor surgery and every patient with clinical stage IV CC as well as every rectal cancer (RC) patient prior to any therapy in a MDT. This recommendation includes that all patients with metastases of colorectal origin should be discussed in a MDT prior to any therapy [2].

## Material and methods

All major institutions providing oncological care to patients in four counties in southern Lower Saxony and Northern Hesse (*N* = 11), subsequently called sample region, were invited to participate in the study. The sample region included urban as well as rural parts and all levels of patient care including a university medical center.

Patients with mCRC and diagnosis of the first metastasis between 01/2011 and 12/2013 were eligible. National demography data including population of the 4 counties in 2011, 2012, and 2013 and raw CRC incidence rate (*www.destatis.de*) were used to estimate the number of CRC patients in the sample region resulting in 1.696 patients for the period 2011–13. Assuming no decrease in incidence rates over the last years and a proportion of 50% developing metastases during the course of disease [6], we calculated that 848 patients with mCRC would represent the maximal sample size for the study. A standardized patient report form (see supplementary material 1) was developed and anonymized data from patient charts were collected and stored in a GCP-conform EDC-system named secuTrial®. The study had been approved by the ethics committee of the University Medical Center Goettingen (AN 23/9/13), the ethics committee of the medical associations in Lower Saxony and Hesse and the data security officials of Lower Saxony and Hesse.

### Preprocessing and statistical analysis

MDT parameter distribution was correlated with clinicopathological parameters (e.g., age, primary tumor localization, number of metastatic sites). The workflow for the preprocessing and the analysis of the data was implemented using KNIME 3.2.1 [7]. KNIME nodes were mostly used for the preprocessing of the data, while we used the R-plugin nodes in KNIME to perform the final statistical tests with R version 3.0.2 [8]. The global significance level was set to *α* = 5%. For comparisons of continuous data we used the Pearson’s correlation coefficient (*r*). If the data were skewed we used the non-parametric, rank based correlation coefficient (tau) according to Kendall [9]. For comparisons of two continuous data distributions we used the Wilcoxon rank sum test; paired where applicable [10]. In case of three or more different distribution samples, we used the Kruskal-Wallis rank sum test for the comparison [11]. In case of count data, we used the Fishers Exact test or chi-square test depending on the available number of samples for the comparison.

## Results

One university medical center, four teaching hospitals, one communal hospital, and 4 oncological focus practices participated in the study. All MDT consisted of at least a medical oncologist, a gastroenterologist, a visceral surgeon with experience in liver surgery and a radiologist. In total, 470 patients (CC *n* = 278, RC *n* = 192) representing 55% of the estimated sample maximum were enrolled.

### Primary tumor therapy

Table 1 summarizes the treatment data regarding primary tumor therapy (considered for metachronous as well as synchronous cases) not necessarily performed within the study period and one of the participating centers. Discussion within a MDT was performed in 141 (63%) of 224 evaluable (data not available for 14 patients) CC patients after primary tumor surgery and in 70 (38%) of evaluable 183 (data not available for 7 patients) patients with clinical stage IV CC prior to any therapy. In RC patients, MDT discussion prior to any therapy was executed in 91 (47%) evaluable patients (data not available in 12 patients). Primary tumor resection was performed in 380 (81%) of 470 patients. To note, 124 of 470 patients were never discussed in a MDT during primary tumor treatment.

**Table 1:** Patient data on primary tumor therapy

Parameter		Colon cancer (*N* = 278)	Rectal cancer (*N* = 192)	All (*N* = 470)
Age^a^ (years)	<70	138	96	234
	≥70	137	96	233
	n/a	3	0	3
	Median	70	70	70
	Range	22–92	29–86	22–92
Sex	Male	159	126	285
	Female	119	66	185
MDT prior to therapy	Yes	79	91	170
	No	179	89	268
	n/a	20	12	32
Neoadjuvant therapy	Yes	-	76	-
	No	-	116	-
Primary tumor resection	Yes	224	156	380
	No	54	36	90
UICC stage	0	0	1	1
	I	8	12	20
	II	28	24	52
	III	54	47	101
	IV	183	106	289
	n/a	5	2	7
MDT post primary tumor resection (*N* = 380)	Yes	141	92	233
	No	69	56	125
	n/a	14	8	22
Adjuvant therapy	Yes	189	134	323
	No	87	56	143
	n/a	2	2	4
MDT never during primary therapy	Yes	78	46	124

^a^Age at the time of primary tumor resection

*Abbreviations: MDT* multidisciplinary tumor board*, UICC* Union International Contre Le Cancer*, n/a not available*

### Treatment of metastases

**Fig. 1:**
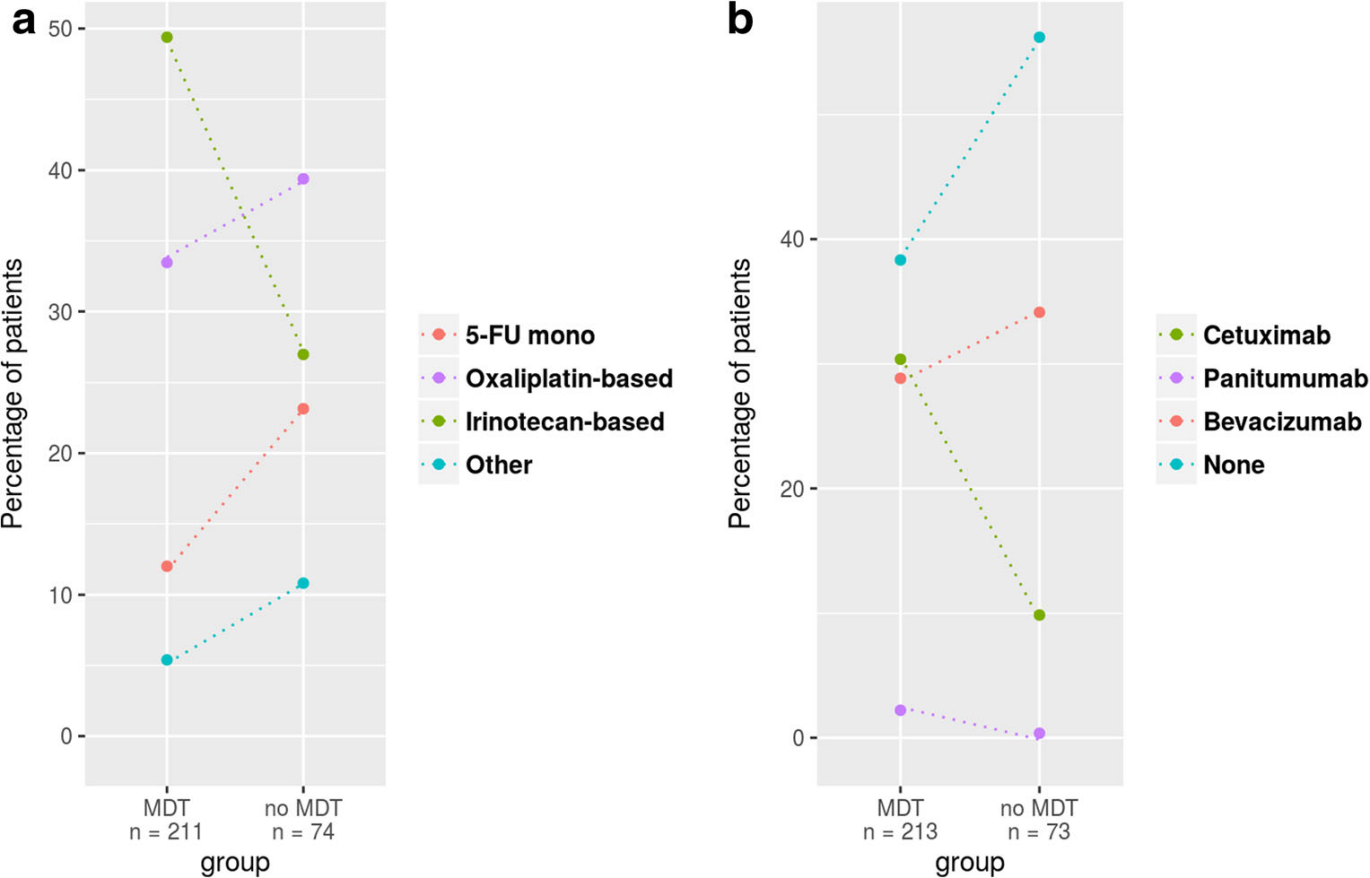
Diversification of chemotherapy backbone (**A**) and administration of monoclonal EGFR/VEGF-antibodies (**B**) according to MDT discussion. Depicted are the relative percentages per group for the different treatment options; *dotted lines* highlight the relative frequency change per treatment regimen observed between the groups. There was a statistically significant difference in both, chemotherapy backbone (*p* = 0.0024) and use of monoclonal antibodies (*p* = 0.0007) between the two subgroups

Table 2 gives a summary of treatment data when hematogenous metastatic dissemination became evident for the first time. Within the metastatic stage of disease, 297 (63%) of all 470 patients were discussed within a MDT (MDT data not available in 11 patients) prior to any therapy. Detailed oncological treatment data on metastases were available for 465 patients. The majority of patients diagnosed with the first metastasis was treated initially by systemic chemotherapy (291 patients, 62%). Overall, there was a significant difference in the chemotherapy backbone used (*p* = 0.0024, Fig. 1A) and addition of monoclonal antibodies (*p* = 0.0007, Fig. 1B) between patients pretherapeutically presented or not presented in a MDT, respectively. Curative resection of metastases was performed in 193 (41%) cases. Of these, 101 (52%) experienced straight forward resection while in 92 (48%) patients, systemic chemotherapy was given preoperatively. Seventy-three (16%) patients received best supportive care only.

**Table 2:** Patient data on treatment of metastases

Parameter		Colon cancer (*N* = 278)	Rectal cancer (*N* = 192)	All (*N* = 470)
Time of metastasis	synchronous	184	102	286
	metachronous	94	90	184
Localisation (overall)	Liver	215	132	347
	Lung	91	96	187
	Lymph node	55	45	100
	Peritoneum	62	22	84
	Brain	10	12	22
	Other	54	43	97
Manifestation	1 organ	186	124	310
	≥ 2 organs	92	68	160
MDT prior to therapy of metastases	Yes	165	132	297
	No	106	56	162
	n/a	7	4	11
First-line chemotherapy	Yes	164	127	291
	+EGFR/VEGF antibody	91	72	163
	No	114	60	174
	n/a	0	5	5
Resection of metastases	yes	109	84	193
	no	169	108	277
MDT post resection of metastases (*N* = 193)	yes	90	65	155
	no	14	13	27
	n/a	5	6	11
Chemotherapy post resection of metastases (*N* = 193)	yes	53	40	93
	no	54	40	94
	n/a	2	4	6

*Abbreviations*: *MDT* multidisciplinary tumor board, n/a not available, *EGFR* epithelial growth factor receptor, *VEGF* vascular endothelial growth factor

**Fig. 2:**
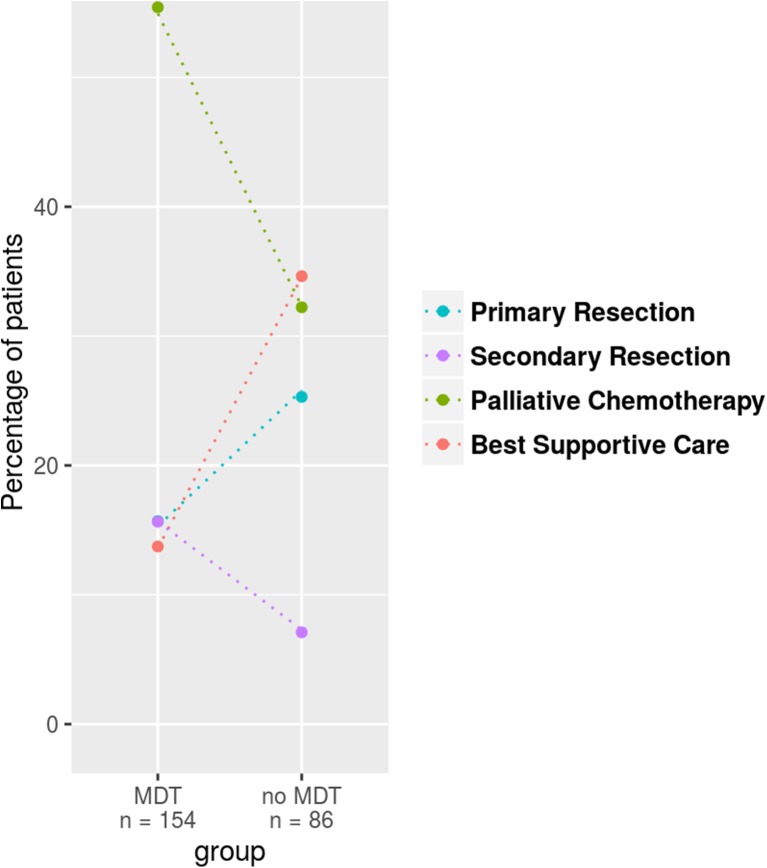
Treatment concepts in patients ≥70 years. Depicted are the relative percentages per group for the different treatment options; *dotted lines* highlight the relative frequency change per treatment regimen observed between the groups. There was a significant difference between the two subgroups with patients presented in MDT receiving more often any treatment in terms of chemotherapy, surgery, or both (*p* = 0.0002)

Patients ≥70 years at the time when metastases had been diagnosed (*n* = 250) received significantly more often any treatment (chemotherapy, surgery, or both) when presented in an MDT (86 versus 64%, *p* = 0.0002, Fig. 2). Although chemotherapy was accompanied by a monoclonal antibody more often in patients presented in an MDT the difference was statistically not significant (54 versus 38%, *p* = 0.1194). Between those patients, ≥70 years that were discussed in an MDT and those that were not, there was no statistically significant difference for the following parameters: sex (*p* = 0.584), UICC stage (*p* = 0.085), timepoint of metastases (*p* = 0.214) and number of metastatic organ sites (*p* = 0.239).

When focusing on patients with single organ metastases the subgroup analysis between those presented in a MDT versus those who were not, showed no statistically different resection rate (48 versus 57%, *p* = 0.1574, Fig. 3) but preoperative chemotherapy was more often indicated (57 versus 33%, *p* = 0.0056) when patients were discussed in a MDT. Between those patients discussed in a MDT and those who were not, there was no significant difference for the following parameters: sex (*p* = 0.717), age (*p* = 0.227), UICC stage (*p* = 0.188) and timepoint of metastases (*p* = 0.402).

**Fig. 3:**
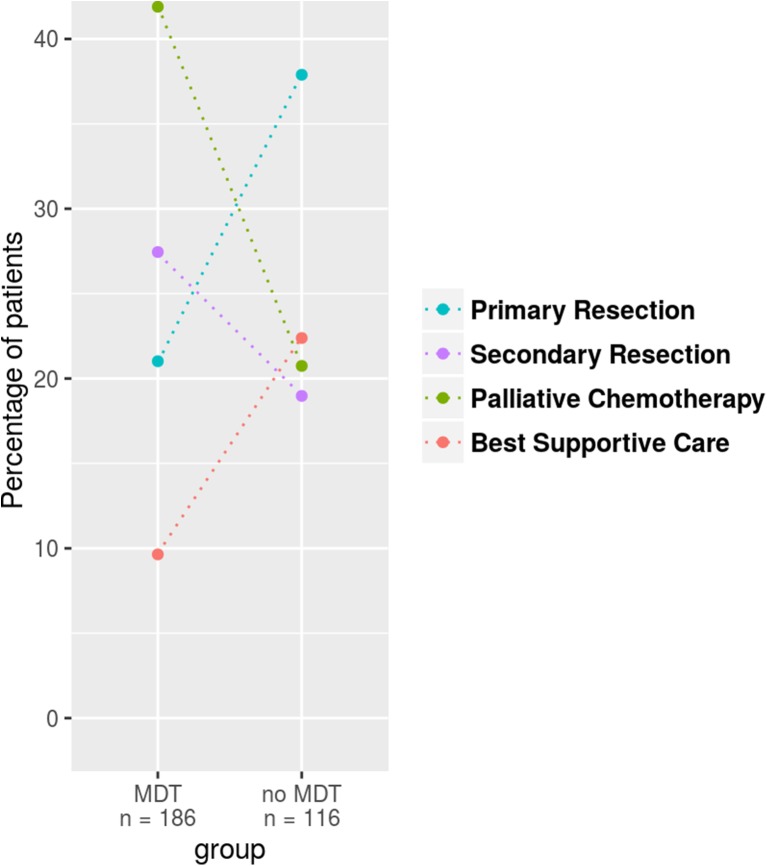
Treatment concepts according to number of metastatic sites in particular focused on those with single organ metastases. Depicted are the relative percentages per group for the different treatment options; *dotted lines* highlight the relative frequency change per treatment regimen observed between the groups. Patients presented in MDT did not receive surgery significantly more often (48 versus 57%, *p* = 0.1574) but focused on operated patients preoperative chemotherapy was more often indicated (57 versus 33%, *p* = 0.0056) when patients were discussed in a MDT

## Discussion

As already stated MDT have been advocated as standard of care in modern oncology even though a direct benefit of MDT discussion in terms of improved patients’ survival has been demonstrated only in few studies [12]. However, previous studies have shown that MDT discussion leads to relevant improvements of interdisciplinary therapy such as more recommendations detached from the case-presenting discipline’s plan [13] and improved adherence to guidelines [14]. On the other hand, healthcare professionals, especially those working in university hospitals and large referral centers criticize feasibility of increasing numbers of time consuming MDT sessions. In this area of conflict, it is of major importance to gain knowledge about MDT utilization in clinical routine.

Different from other European countries and the US, the initiation of a nationwide clinical cancer registry has started not earlier than 2013 when the German Government adopted the *Law for Early Cancer Diagnostics and Quality Assurance by Clinical Cancer Registries* [15]. The corresponding standard report form includes notification of MDT date and timepoint of discussion (pretherapeutical, postoperative, or posttherapeutical) [16]. Unfortunately, the federal states did not harmonize their individual databases until today hampering nationwide analyses on MDT utilization on the basis of such registries. Therefore, the results of this study are of major interest.

Given the mentioned benefit of MDTs in previous publications as well as the significant differences in treatment decisions between MDT and non-MDT discussed cases as observed within this study, the overall low MDT utilization in all analyzed clinical scenarios in the present study raises relevant concerns. With 55% of estimated cases in the sample region involving most major players and a sample region layout applicable to many regions nationwide, it has to be assumed that comparable results would be obtained when other regions in Germany were investigated. As all participants in this study had access to at least one MDT, we can only hypothesize on the surprisingly low rate of MDT discussion in mCRC. One reason might be the supposed clarity of the clinical scenario in individual patients with clear guideline recommendations or strong conviction of the treating physician for the superior treatment and subsequent perceived needlessness for multidisciplinary discussion. Such thinking is underlined by our previous survey on experienced medical oncologists and surgeons who mainly stated that they were able to access correctly the complementary specialties’ treatment options in patients with mCRC [17]. However, there are prominent examples that even highly specialized experts do not homogeneously assess individual patient cases [18]. Very recently it has been shown by evaluation of 213 rectal cancer cases that MDT discussion led to a change in treatment schedule in 70 (33%) cases including 22 cases (10%) in which the presenting physician had a “definite plan” prior to MDT [19]. Furthermore, the ongoing elucidation of molecular subtypes in colorectal cancer [20] as well as treatment relevant clinical parameters such as localization [21] make even formerly simple clinical scenarios more and more complex requiring involvement of all available expertise in every treatment decision which can most easily be done within a MDT discussion. Another potential reason could be that single institutions actually never or only in selected scenarios discussed their patients within a MDT. This cannot be excluded because as part of the study policy the patient data were not linked to any institution code while transferred to the data base precluding inter-institutional comparison. However, the overall high number of patients not discussed in a MDT is unlikely generated by a single institutions performance.

Focusing on consequences of MDT utilization particularly in patients older than 70 years, it becomes obvious, that waiving the MDT discussion was not accompanied by restriction of therapy in general but less application of chemotherapy (Fig. 2). There is a huge discussion about how much therapy should be applied to older patients and it has been shown in other studies that the older patients are the less chemotherapy they get [22, 23]. However, biological age is not a straightforward parameter to trigger such treatment decisions. Unfortunately, data on ECOG status were not available for the majority of patients in our study, so other than planned any analysis considering ECOG status could not be done which would have been of great interest especially in older patients.

Once accepting that physicians do not inherit enough intrinsic motivation to discuss all their patients in MDT, other strategies to overcome the low rate of MDT discussion need to be developed. One of these could be a certification program for colorectal cancer centers which in Germany has been implemented by the German Cancer Society (DKG) beginning in 2006. Besides numerous other key figures monitored, the DKG has set the rate of pretherapeutical MDT discussion in RC patients to a minimum of 95%. From 2009 to 2011 this median rate of pretherapeutical MDT discussion in RC patients in all certified centers raised from 88.0 to 91.8% [24] showing two facts: first, certification resulted in higher MDT discussion rates than those observed in our sample region but second, even these certified centers did not meet the minimum requirements. Of note, none of the participating institutions in our study had been one of the 247 DKG-certified colorectal cancer Centers approved until 2013.

Concerning limitations, the results of this study might be biased by its retrospective design and related lack of MDT documentation as data were collected by chart review only. Furthermore, the study was not designed to evaluate a potential difference between the MDT recommendation and the actual treatment of patients. The reason for that is that every MDT had its own structure and some prefer to include patients’ preferences into the discussion process while others favor an interprofessional discussion first followed by a separate discussion on the recommendation with the patient as advocated by the British NICE guidelines [4]. In addition, the growing acceptance of MDT in general within the last years might have already resulted in an increased MDT utilization in the sample region and other parts of the country since 2013.

In conclusion, in clinical routine MDT discussion is not as established as advocated by national guidelines. Treatment decisions differ depending on MDT discussion especially in older patients and those with single organ metastases. Given the demonstrated benefits of MDT discussion concerning patient survival, efforts should be made to improve cancer care in terms of MDT utilization for which certification programs can be a reasonable option.
